# Comparison of Diploid and Triploid Atlantic Salmon (*Salmo salar*) Physiological Embryonic Development

**DOI:** 10.3390/ani13213352

**Published:** 2023-10-28

**Authors:** Callum Howard, John F. Taylor, Herve Migaud, Alejandro P. Gutierrez, Michaël Bekaert

**Affiliations:** 1 Institute of Aquaculture, University of Stirling, Stirling FK9 4LA, UK; 2 AquaBioTech Group, 1761 Mosta, Malta; 3 AquaMaof Aquaculture Technologies Ltd., Rosh Ha’ayin 4809245, Israel; 4 Mowi Scotland, Glen Nevis Business Park, Fort William PH33 6RX, UK; 5 Center for Aquaculture Technologies, San Diego, CA 92121, USA

**Keywords:** aquaculture, ploidy, *Salmo salar*, developmental stages, timing to hatch, mortality

## Abstract

**Simple Summary:**

This study looked at the development rate from the moment of fertilisation to hatching in both diploid and triploid Atlantic salmon. These two types of salmon have distinct physical and biological characteristics, including differences in their hearts, brains, digestive systems, their ability to handle different temperatures as eggs and after hatching, and their nutritional needs. The study did not find a significant difference in the rate at which the two types of embryos developed their physical characteristics. However, there were two notable differences between the two types of salmon. Triploid salmon hatched earlier than diploid salmon and reached the halfway point of hatching significantly sooner. Unfortunately, triploid salmon also experienced a higher rate of mortality. These findings provide valuable insights for future research involving Atlantic salmon aquaculture.

**Abstract:**

Diploid and triploid Atlantic salmon show distinct physiological differences including heart, brain, and digestive system morphology, propensity for certain deformities, temperature tolerance as eggs and once hatched, and different nutritional requirements. Whilst several studies have looked in detail at the rate of embryogenesis in diploid salmon, no study has compared the rate of embryogenesis between ploidies from fertilisation to hatch. This study based its assessment on a seminal paper by Gorodilov (1996) and used the same techniques to compare the rate at which triploid and diploid embryos developed morphological characteristics. Whilst no significant difference was found, this study provides well-needed justification for the assumption that both ploidies develop at the same rate and gives scientific weight to studies which involve manipulation at these stages of development. Two factors that did differ, however, were the timing of hatch, and mortality. Triploids hatched more quickly than diploids and reached 50% hatch at a significantly earlier point. Triploids also suffered from a significantly higher rate of mortality.

## 1. Introduction

Atlantic salmon (*Salmo salar*) triploids, having three sets of chromosomes, are presented as a functionally sterile alternative to traditional diploids, potentially diminishing the ecological effect of escapes on wild populations and removing the costs associated with early maturation. Triploidy in salmon inhibits the second meiotic division, yielding sterile fish [1]. Triploidy also results in a fish with fewer but larger cells [2]. The differences between triploid and diploid Atlantic salmon extend beyond these basic characteristics, which explains the limited industry acceptance in areas where early maturation is an issue (e.g., Tasmania [3]) or where sterility is a licensing requirement (e.g., New Brunswick, genetically engineered salmon, and formerly in Norway under Green licences). These differences include various aspects such as gill filament density [4,5], as well as gut [6], brain [7,8], and heart morphology [9,10]. Triploids tend to display a higher susceptibility to a variety of deformities, including those affecting the vertebrae [11], lower jaw [12], eyes [13], and heart [10]. They require lower incubation temperatures [10,14,15] and ploidy-specific diets with increased histidine [13,16,17], phosphorus [11,18,19], and protein [18].

Despite the differences between the two ploidy levels, little research has been undertaken to explore the potential impact of ploidy on the rate of embryogenesis. Extensive research has been conducted on the embryogenesis rate in diploid Atlantic salmon, including a notable study by Gorodilov [20] describing the rate at which physiological characteristics developed, the transition between developmental stages, and considering the role of temperature. The timing of development was assigned the denominator Tau somite, that is, the rate at which one somite pair forms at a given temperature. Gorodilov’s work has since been used in numerous subsequent experiments to accurately time treatments to specific developmental stages—an essential aspect of research that lends credibility to scientific discoveries and facilitates comparison between different experiments [20].

Despite the critical nature of this issue, only two studies have sought to explore the embryogenesis rate in triploid Atlantic salmon [21,22]. Both studies were limited in their scope. In the study conducted by Johnston et al. [21], the rate of embryogenesis was not the primary focus and only the somite formation rate was compared; no difference between ploidies was found at this stage of development. Intriguingly, the somite pair formation rate reported in Johnston et al. [21] diverged from Gorodilov’s findings; this may have resulted from the genetic origin of the individuals used. In the study in Gray et al. [22], only two stages of early development were compared. This study also found no difference between triploids and diploids of four other salmonid species at the same stages; the study did, however, use a heat shock to induce triploidy and saw high rates of deformity, likely due to a combination of shock type and incubation at 11.5 °C. Whilst these studies concluded that no difference in the rate of embryogenesis existed before or during somitogenesis, they did not consider developmental stages after somitogenesis. A small number of studies have reported differential hatch rates between triploid and diploid salmonids [23,24], suggesting potential differences in the rate of embryogenesis, at least at the later stages of development. Due to the lack of studies at later stages of embryonic development, assumptions regarding the synchronised parallel development of triploids and diploids across the whole of embryogenesis are unverified. This lack of confirmation raises concerns about the potential for inaccurate staging of experimental treatments based on fertilisation timing, dependent on ploidy. If the rate of embryogenesis does indeed differ at the later stages of triploid development, then uncertainty is introduced into studies comparing differences between ploidies at these stages.

The aim of this study was to compare the embryogenesis rate of triploid and diploid Atlantic salmon, from fertilisation to hatching. We used Gorodilov’s study [20] as a blueprint to identify the transition points between developmental stages. Our goal was to ascertain whether the developmental timing disparities extend to embryonic stages as a function of ploidy.

## 2. Materials and Methods

### 2.1. Experimental Design

A total of 6000 green eggs were stripped from 2 hens at AquaGen Holywood facilities, Scotland. Milt from 1 male was also taken. The eggs and milt were taken to the University of Stirling, where equal volumes of eggs were mixed and split by future ploidy. All eggs were then fertilised with an excess of milt. After fertilisation, the eggs were rinsed with 8 °C water and left to harden in an 8 °C water bath for half an hour. The “triploid” batch of eggs was placed into a pressure vessel and shocked 37 min post-fertilisation using a hydrostatic pressure of 9500 PSI (65,500,000 pascals) for 6 min and 15 s [18]. Triploidy was verified using the microsatellite panel previously developed [25]. A total of 250 eggs from the triploid batch were collected at 150 degree days (DD), and DNA was extracted and sent for analysis in 96-well plates.

After shock, the eggs were removed from the pressure container and allowed to water-harden for 1 h before 3000 triploids and 3000 diploids were laid down across 6 egg trays per ploidy (500 eggs per tray). These eggs were placed into an incubator running at 6 °C ± 0.1 °C. The eggs were checked twice daily and any dead eggs removed. A 30% water change was conducted 3 times a week, using de-chlorinated water at the same temperature as the incubator.

Every 3 days, 20 eggs were taken for each ploidy. The eggs were cleared by placing them into a beaker of ethanol:formalin:glacial acetic acid (6:3:1) for 20 min. The eggs were then placed into a 35 mm Petri dish and observed under a dissecting microscope and photographed. To minimise reflection on the egg, the Petri dish was mostly filled with a 6:1 solution of ethanol and glacial acetic acid whilst being photographed. During somitogenesis, photos of 66 eggs per ploidy were taken every 3 days. After somitogenesis, the number of photos decreased back to 20 per ploidy. The photographs were compared to each other and detailed drawings in Gorodilov [20] and assigned a stage of development from 1 to 34. The value was then used to determine whether there were any differences in the rate of development between ploidies. This approach continued until hatch, at which point the experiment was terminated. Over the period of hatch, hatched alevins were removed from each trough daily and recorded; this continued until all the eggs hatched or died. Embryos before, during, and after somitogenesis were also removed from the egg using micro-dissection and observed under a dissecting microscope to determine if this method was reliable at producing a clearer picture.

### 2.2. Statistical Analysis

When comparing the rate of embryogenesis and mortality at hatch, Levene’s test was used to test for equality of variance using SPSS statistics v26 (IBM Corp, Armonk, NY, USA). The rate of embryogenesis was further analysed using Mann–Whitney *U*-Test to determine if there was a statistically significant difference between the stages of embryogenesis of each ploidy at each sampling point (*n* = 3). To account for the increased risk of type 1 error, the Holm–Bonferroni correction was used to adjust for multiple comparisons. Differences in mortality between ploidies were assessed using Mann–Whitney test on cumulative percentage mortality. SPSS was also used to run a log rank test to determine if there was a difference in the rate of hatch between diploids and triploids.

## 3. Results

### 3.1. Triploidy Validation

Triploid identification was performed using a suite of nine microsatellites. This suite gave a 95.58% triploidy confirmation, but the suite was unfortunately limited in its coverage, and it is possible that type 2 error reduced the percentage confirmation.

### 3.2. Development Stages

**Figure 1:**
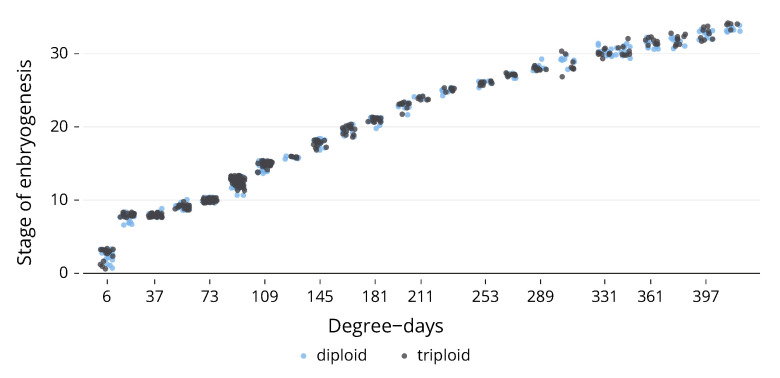
Average stage of embryogenesis between ploidies. Stages as determined by Gorodilov [20].

After all photos were taken, they were compared to each other and to the detailed drawings found in Gorodilov [20]; these comparisons were used to determine the stage of embryogenesis from 1 to 34 (Figure 1). Examples of diploid and triploid photos can be found in Figure S1, alongside the drawings of the matching stage from Gorodilov [20].

**Figure 2:**
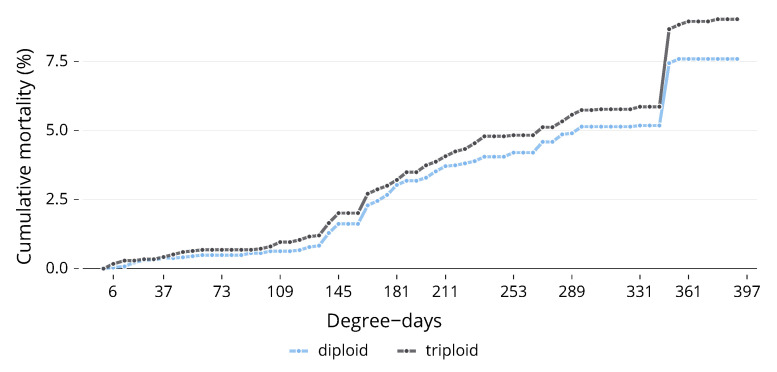
Cumulative mortality (%) by day of triploid and diploid Atlantic salmon (*Salmo salar*). Eggs incubated at 6 °C.

Once all the photographs had been assigned a stage of embryogenesis, they were compared to determine if there was a difference between ploidies. No significant differences were found at any sampling point up until hatching began (Figure 1). There was a significant (U = 1445, *p* < 0.001) difference in mortality between ploidies; triploids had a total mortality of 19.89% by the end of the experiment, compared to 10.93% for diploids (Figure 2).

All ploidy troughs showed a slight increase in mortality around 133 DD. Both ploidies showed a gradual increase in mortality from this point until 349 DD; this corresponds to the fact that at 337 DD, all treatments were physically shocked to remove non-viable eggs (this is commercial standard practice). Mortality reached 9% and 7.6% for the triploid and diploid, respectively.

### 3.3. Hatching

**Figure 3:**
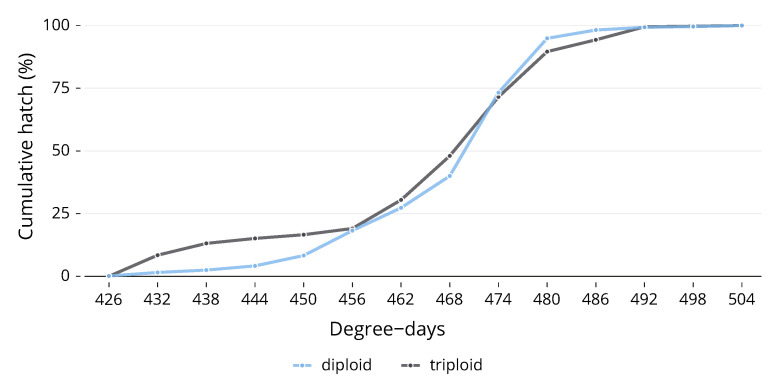
The hatching distributions for the two ploidies to 50% hatch were statistically significantly different.

Due to the fact that, as alevin, it was impossible to completely separate each of the egg trays in the trough, the number of replicates for hatching was reduced from 3 to 1, as the whole trough was counted together. Whilst there was no significant difference between ploidies in their development during embryogenesis, there was a difference in the pattern of hatch. Diploids experienced the first hatch with one hatching on 426 DD. After this point, the number of triploids hatched rose more rapidly than the diploids, and over the next few days, the cumulative hatch of triploids was higher than that of diploids (Figure 3). On the fourth day of hatch (444 DD), 15% of total triploids had hatched, whilst only 4% of diploids had hatched. Diploids began to catch up after this point, although by the eighth day (468 DD), 48% of triploids had hatched compared to 40% of diploids. The gap closed after this point, and both ploidies reached 100% hatch on the same day. The hatching distributions for the two ploidies to 50% hatch (Figure 3) were statistically significantly different, ($\chi^{2}$ (1) = 12.141, *p* < 0.0005, *n* = 1). There was no significant difference in time to 100% hatch.

## 4. Discussion

Our study reveals no significant disparity in the embryogenesis rate between triploid and diploid Atlantic salmon until hatching occurs. The developmental stage, based on Gorodilov’s established model [20], did show no differential in ploidy at any given time. This suggests that while triploidy profoundly impacts numerous physiological attributes, it seemingly does not alter the embryogenesis rate.

Even in the early developmental stages characterised by two and four cells, we noticed no variance between ploidies, although it should be stated that the observational resolution of these early stages could be improved, as samples were collected every 3 days. In agreement with Johnston et al.’s [21] conclusion, our study found no variation in the somitogenesis rate between ploidies, and this held true for the stages prior to and following somitogenesis.

Although no differences were noted when embryos reached specific stages during embryogenesis, a distinct pattern emerged during hatching. As previously observed in triploid rainbow trout [23,24], triploid Atlantic salmon during the current experiment hatched earlier, albeit by a minor degree day margin. This could indicate subtle developmental differences during the later stages of embryogenesis, though these did not manifest as overall developmental stage differences. Potential explanations for this phenomenon include a slower mitotic rhythm in triploids due to larger cell sizes [24], or the possibility that fewer cells per organ [2] may reduce the number of mitotic cycles required during embryogenesis. This balance could contribute to a slightly increased embryonic rate in triploids [24]. However, these propositions require further exploration, as our current study and two previous studies [21,22] found no variations in embryonic development rates. Another possible explanation involves the increase in heterozygosity. Both rainbow trout and Atlantic salmon have shown a positive relationship between heterozygosity and developmental rate, after hatching [26,27], pre-first feeding [28], and for the timing of hatch [29,30]. Triploid Atlantic salmon, having inherited an extra set of maternal chromosomes, exhibit greater heterozygosity than their diploid counterparts [31,32]. The relationship between heterozygosity and growth rate is complex, with the specific genetic mechanisms involved playing a crucial role [29].

The size of the fish at hatch was not investigated in this study; triploids typically hatch smaller than diploids [33] and show higher rates of mortality and deformities after hatch, with a decrease after first-feeding [34]. Whilst a differential pattern of hatch has not been reported in previous experiments on Atlantic salmon, the smaller size at hatching and higher rate of early mortality could be linked to earlier or faster rates of hatching, as seen in the current experiment. Faster rates of hatch could lead to individuals who are developmentally unready due to hatching prematurely. The relationship between hatching size and the differential pattern of hatch observed in this study merits further research.

Our mortality data showed a slight increase at 133 DD (approximately 27 somite pairs) and 157 DD (approximately 40 somite pairs), regardless of ploidy. These stages coincide with key developmental milestones, such as neuromere formation in the hindbrain and heart tube appearance at around 133 DD, and gill region segment formation and heart tube bending at around 157 DD [20]. Minor disruptions in these crucial processes could account for the observed minor increase in mortality.

Despite these two peaks, mortality remained relatively stable, increasing slowly until the eggs were manually shocked, causing non-viable eggs to whiten. Once these eggs were discarded, mortality did not rise until hatching. Our observed mortality did not deviate significantly from previous literature. Clarkson et al. [14] reported a mortality of 16.4% ± 19.2% for diploids and 22.3% ± 21.9% for triploids from fertilisation to 400 DD at 6 °C, compared to 10.93% and 19.89%, respectively, for the current experiment. The reasons for the higher rate of mortality seen in triploids during embryogenesis are multifaceted. Triploids tend to suffer increased mortality at higher incubation temperatures [14,15]. Triploids also suffer from increased sensitivity to egg quality [33]. One also cannot rule out the impact of the triploidy induction process itself, either due to stress or the increased rates of chromosome aberrations that the process can cause [35].

## 5. Conclusions

In conclusion, we found no differences in the embryogenesis rate between diploid and triploid Atlantic salmon, although a difference was observed in the hatching pattern. There was a significant difference in the mortality between ploidies up until hatching, with triploids suffering from a higher rate. This study expands on previous studies and confirms that the rate of embryogenesis does not differ between ploidies, allowing future experiments to be conducted in the confidence that treatments during embryogenesis will be conducted at the same developmental stage on both ploidies. The differential pattern of hatch poses interesting questions as to the reason behind the faster hatch rate in triploids.

## Data Availability

All relevant data are within the manuscript and its Supplementary Materials.

## Supplementary Materials

The following supporting information can be downloaded at: https://www.mdpi.com/article/10.3390/ani13213352/s1, Figure S1: Comparison of Diploid and Triploid Atlantic Salmon (*Salmo salar*) embryos.

## Abbreviations

The following abbreviation is used in this manuscript:

DD	Degree Days
