# Supplementary material for: Comparison of Diploid and Triploid Atlantic Salmon (Salmo salar) Physiological Embryonic Development
Source: Animals (Basel). 2023 Oct 28;13(21):3352. doi: 10.3390/ani13213352 (PMC10647732; doi:10.3390/ani13213352)
Supplement: Supplementary file 1 [file animals-13-03352-s001.zip › animals-2648674-supplementary.pdf]

Supplementary Materials: Comparison of diploid and triploid Atlantic salmon (*Salmo salar*) physiological embryonic development

Callum Howard, John F. Taylor, Herve Migaud, Alejandro P. Gutierrez and Michaël Bekaert

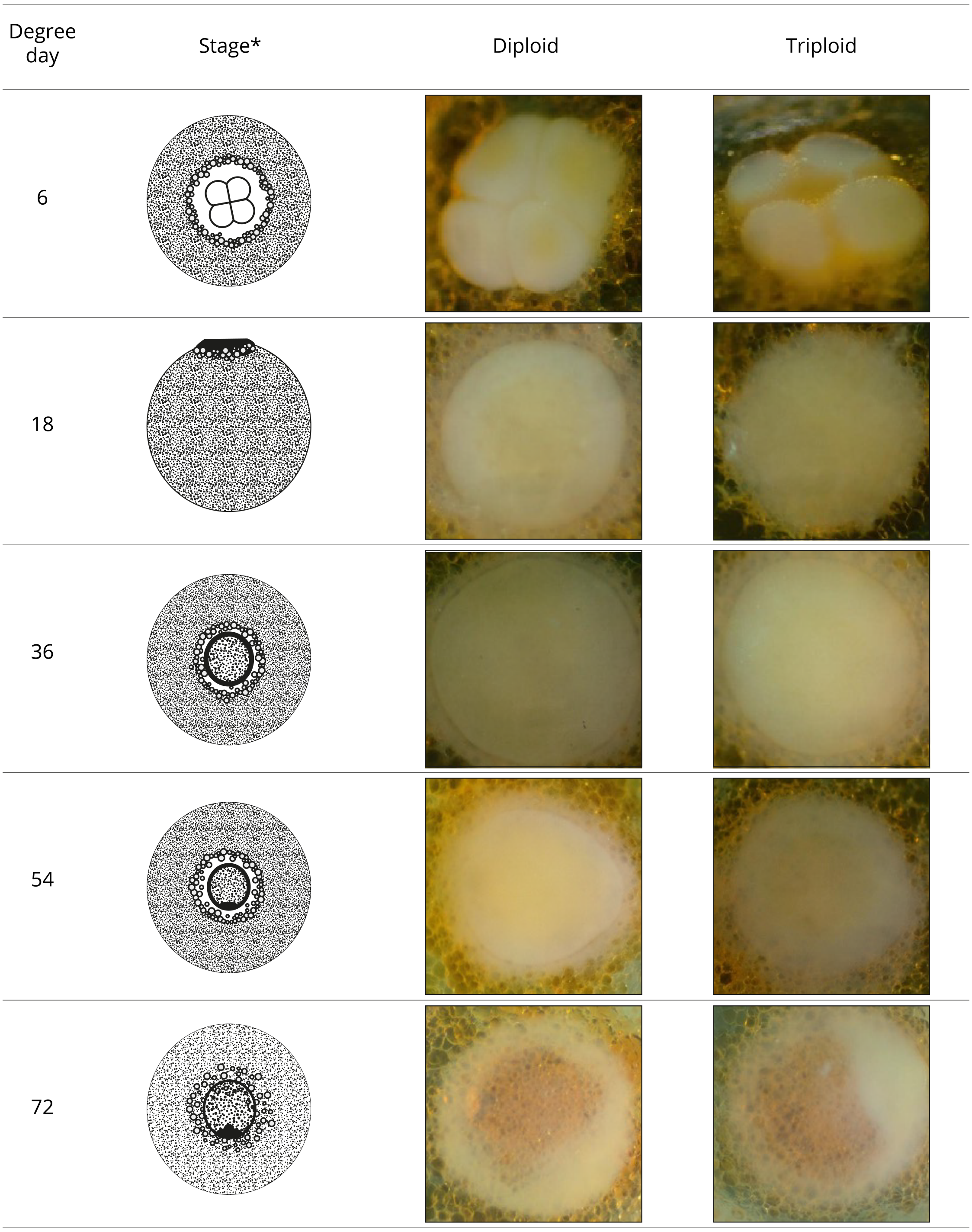

Figure S1. Comparison of Diploid and Triploid Atlantic Salmon (*Salmo salar*) embryos to the drawings of diploid embryos found in Gorodilov [1]. Degree-days state the time at which the photographs were taken.

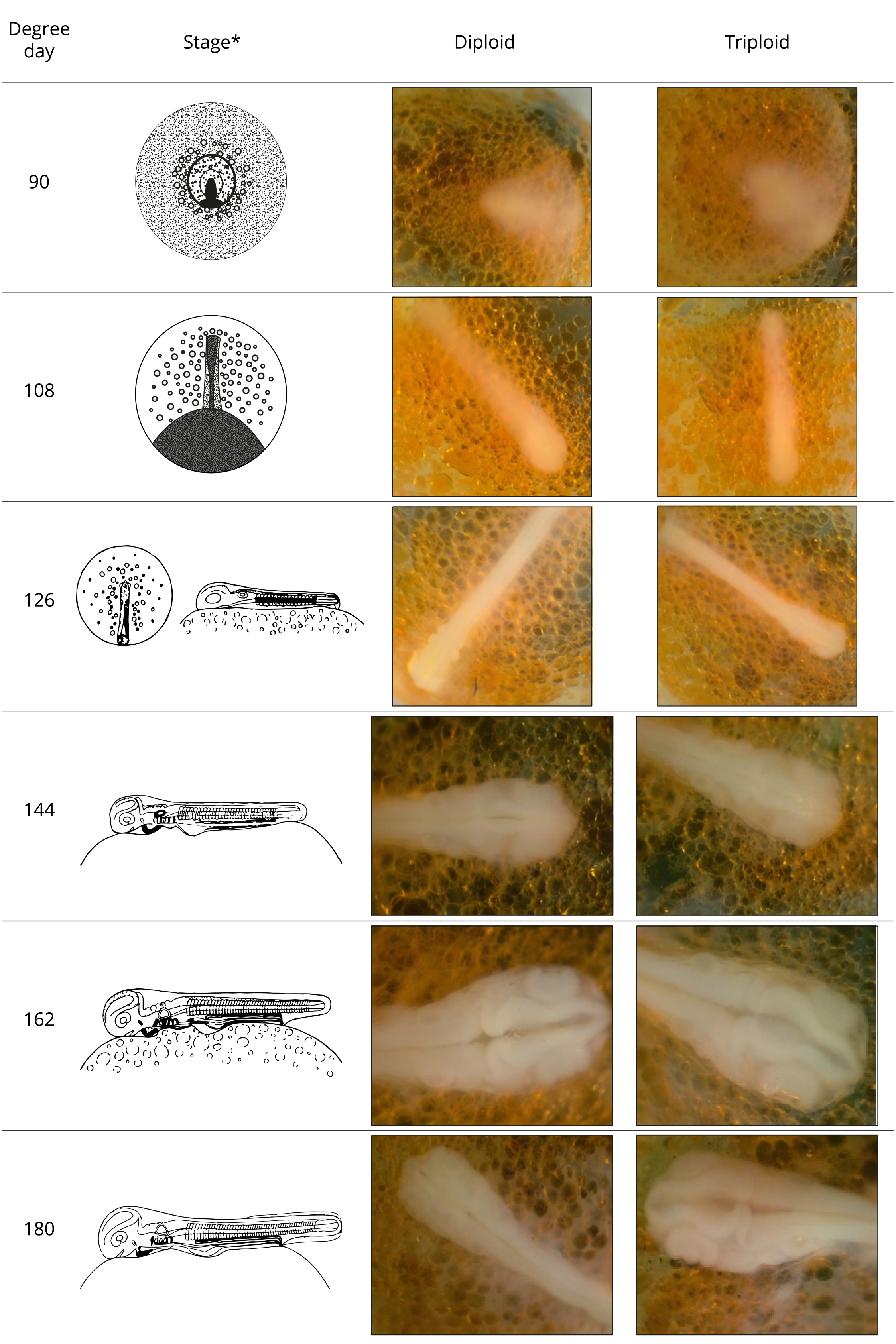

Figure S1. Degree day 90 to 180.

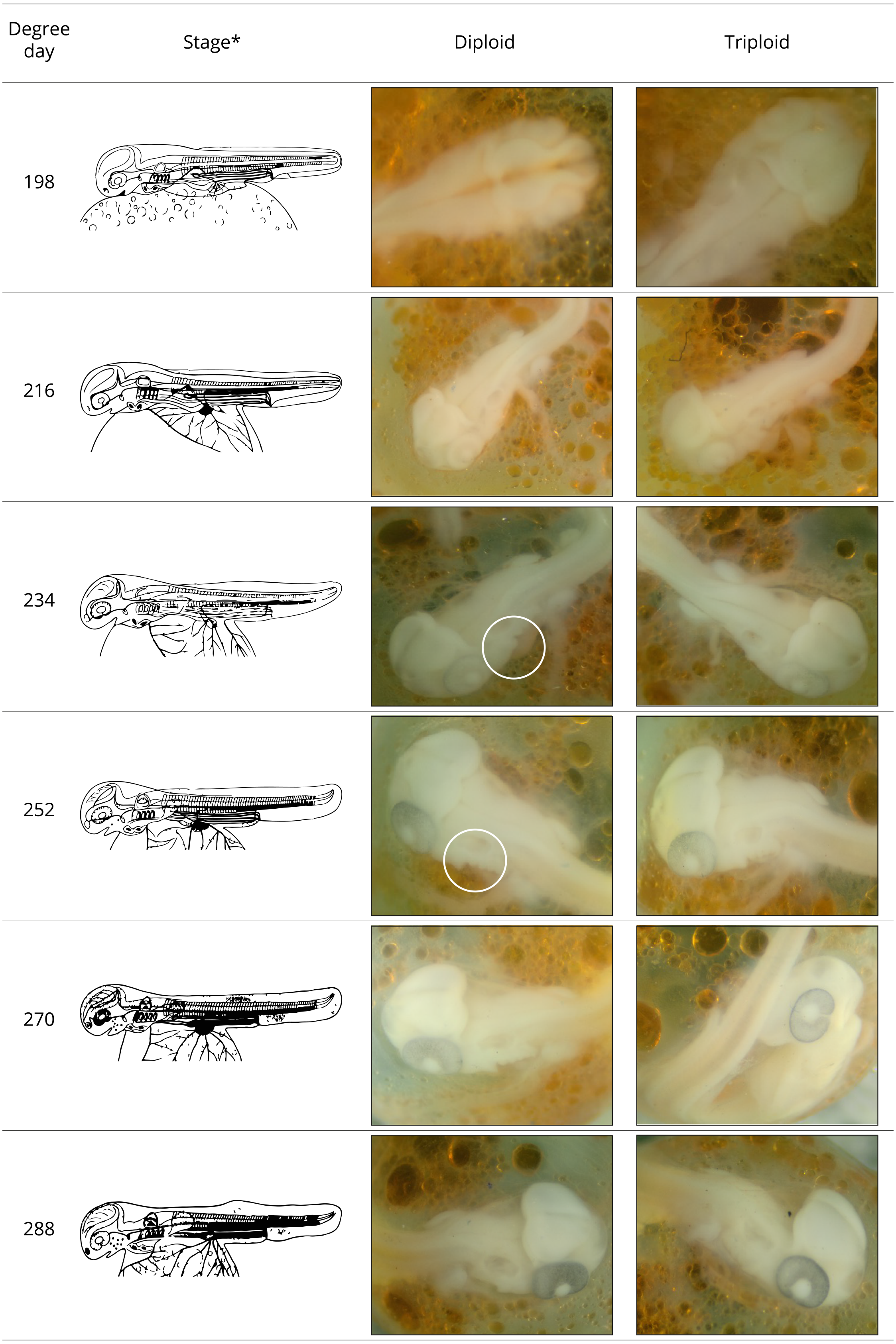

Figure S1. Degree day 198 to 288. For clarity gill arches are highlighted by a circle in pictures at 234 and 252 degree days, they can also be observed in a number of other photo.

Degree day	Stage*	Diploid	Triploid
306	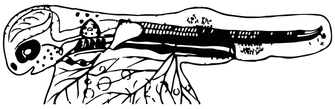	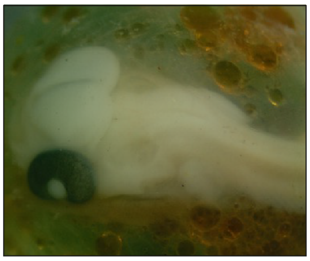	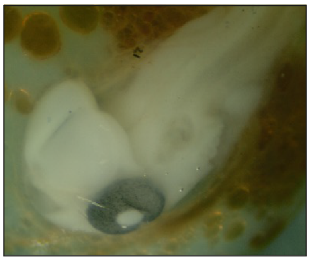
324	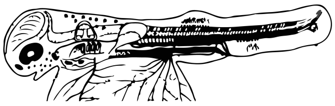	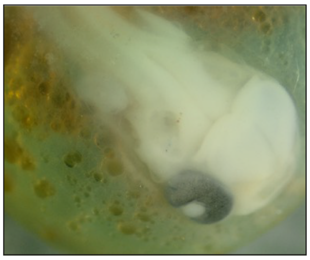	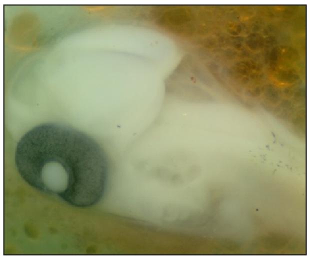
342	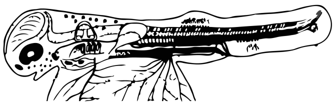	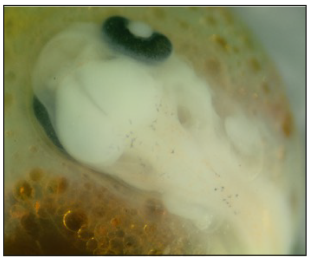	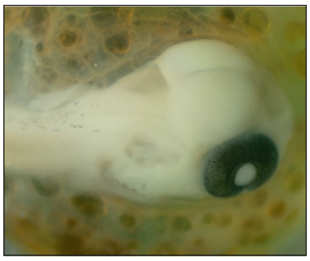
360	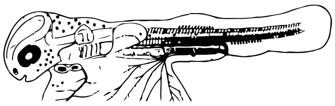	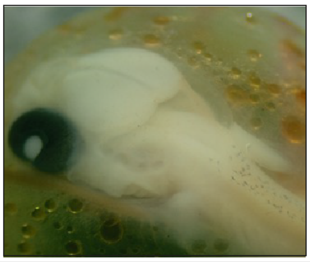	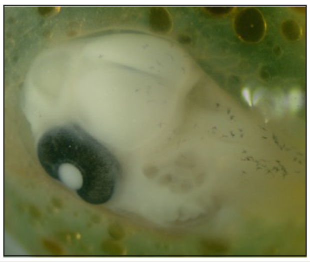
378	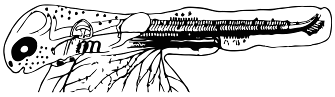	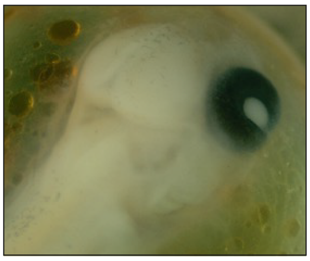	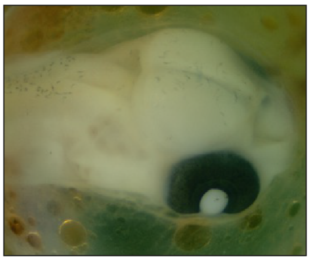
396	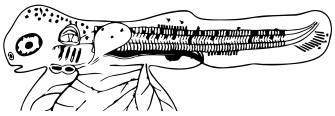	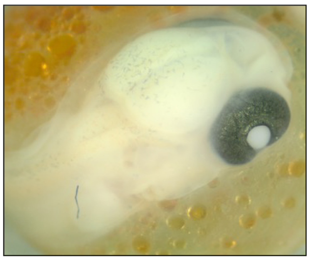	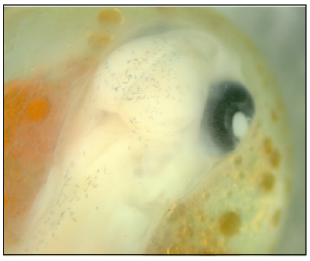

Figure S1. Degree-day 306 to 396.

Degree day	Stage*	Diploid	Triploid
414	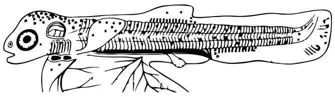	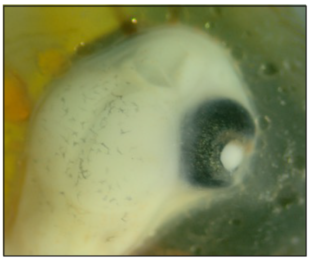	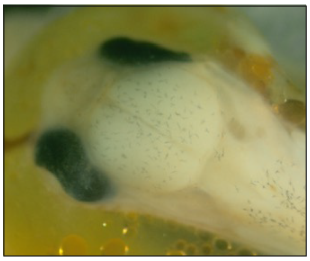
432	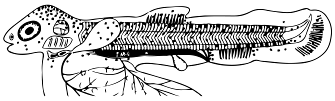	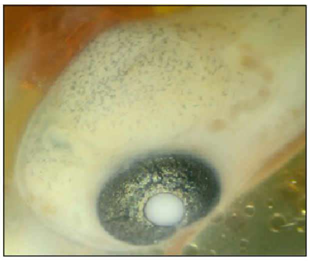	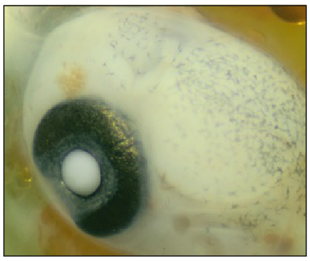

Figure S1. Degree-day 414 to 432.

References

1. Gorodilov, Y.N. Description of the early ontogeny of the Atlantic salmon, Salmo salar, with a novel system of interval (state) identification. *Environ. Biol. Fishes* **1996**, *47*, 109–127. https://doi.org/10.1007/bf00005034.

Disclaimer/Publisher's Note: The statements, opinions and data contained in all publications are solely those of the individual author(s) and contributor(s) and not of MDPI and/or the editor(s). MDPI and/or the editor(s) disclaim responsibility for any injury to people or property resulting from any ideas, methods, instructions or products referred to in the content.
